# Methods for Rapid and Smart Characterization of Vanadium Oxide Thin Films and Thermally Aged Samples Using Raman Spectroscopy and Multivariate Analysis

**DOI:** 10.3390/s26196308

**Published:** 2026-10-06

**Authors:** Logan Echeveria, Sarah Sahota-Dhillon, Tyler Fears, Alyssa Maich, Amelia Petersen, Tiziana Bond, Scott Donald

**Affiliations:** Lawrence Livermore National Laboratory, Livermore, CA 94550, USA; sahotadhillo1@llnl.gov (S.S.-D.); fears1@llnl.gov (T.F.); maich1@llnl.gov (A.M.); petersen37@llnl.gov (A.P.); bond7@llnl.gov (T.B.); donald3@llnl.gov (S.D.)

**Keywords:** Raman spectroscopy, vanadium oxide, principal components analysis

## Abstract

Vanadium oxides (VOxs) are technologically important materials because of their versatile properties, including a reversible metal-insulator transition. However, the complex vanadium-oxygen phase diagram includes numerous stable stoichiometries and metastable polymorphs, making material identification challenging. This work presents a data-driven, nondestructive characterization workflow that combines Raman spectroscopy and principal component analysis (PCA) to provide rapid and intelligent feedback during VOx thin-film fabrication. Although Raman spectroscopy provides ultrasensitive chemical information, conventional spectral interpretation requires specialized expertise and extensive reference data. An in-house fabrication protocol based on spin-casting chemical precursors and thermal annealing was developed to produce thin-film standards of VO_2_, V_2_O_5_, V_3_O_7_, and V_6_O_13_. Raman fingerprints from these standards were used to construct two- and three-dimensional PCA models that distinguish stoichiometric variation and provide interpretable feedback during fabrication-process development. The standards also serve as references for evaluating thermally corroded samples. These results demonstrate that PCA-enabled Raman analysis can serve as an adaptive sensing approach for rapid assessment of VOx stoichiometry, simplified spectral interpretation, and fabrication optimization.

## 1. Introduction

Vanadium oxides (VOxs) have unique physical and chemical properties that have led to their adoption across many industrial and sensor-based applications. Most prominently, V_2_O_5_ is gaining momentum as a lithium-ion battery cathode material due to its high theoretical specific capacity of approximately 450 (mA h)/g [1,2]. As one of the most stable and widely used VOx phases, V_2_O_5_ is also investigated for applications that leverage its strengthening and catalytic properties. Another attractive feature of VOxs is their temperature-induced, reversible change in electrical conductivity and infrared reflectivity. Specifically, VO_2_ undergoes a reversible metal-insulator transition at 68 °C [3]. The ability of the material to switch between a metallic and insulating state has led to the development of VO_2_-based thermochromic smart windows that can significantly reduce energy consumption in residential buildings [4]. This property has also been observed in other VOx phases and has historically supported the development of VO_2_-based electronic and infrared optical switches, supporting a multitude of sensor fields such as temperature, gas, chemical, and pressure, and biological sensors. [5,6] For example, it was recently shown that machine-learning-optimized, tungsten-doped VOx multilayers can enhance infrared microbolometer sensitivity by providing a large, linear resistance response to temperature changes [7].

Apart from these specific applications, VOx materials exhibit a high number of accessible vanadium oxidation states, including V^5+^, V^4+^, V^3+^, and V^2+^, as well as several stoichiometric mixed oxides, such as V_6_O_13_, V_3_O_7_, and V_3_O_4_. For many decades, this key characteristic has been exploited in the use of VOx catalysts for chemical manufacturing processes, such as sulfuric acid production, environmental-pollutant reduction, and the removal of nitrogen oxides and carbon monoxide from industrial emissions [8,9,10]. This versatile oxidation behavior is beneficial for redox applications but also creates a highly complex challenge when characterizing vanadium under oxidizing conditions. For example, the vanadium-oxygen phase diagram features a multitude of stable oxides, numerous metastable phases, and highly sensitive phase boundaries [11,12]. Therefore, robust diagnostic tools are needed to accurately classify oxide stoichiometry and thickness.

State-of-the-art diagnostic tools, such as grazing-incidence X-ray diffraction (GI-XRD) for phase identification and cross-sectional transmission electron microscopy (TEM) or focused ion beam scanning transmission electron microscopy (FIB-STEM) for thickness analysis, are either time-intensive or destructive. Raman spectroscopy offers many advantages over these techniques: it is a well-established fingerprinting technique that requires minimal sample preparation, enables rapid acquisition, and is nondestructive. It operates by measuring wavelength shifts in laser light that is inelastically scattered through interactions with the sample’s characteristic molecular or lattice vibrations. Other nondestructive methods, such as X-ray photoelectron spectroscopy (XPS), require complex alignment or vacuum equipment and therefore impose a greater time burden. However, Raman spectra can be complex and typically require a well-established reference library or expert analysis for proper interpretation.

To address this limitation, this work integrates the chemical sensitivity of Raman spectroscopy with principal component analysis (PCA) to resolve systematic patterns in complex VOx spectra. Unlike conventional approaches that rely on interpreting Raman peaks through spectral-library comparisons, which can become challenging when spectra contain overlapping features or contributions from multiple chemical species, the proposed Raman-PCA workflow simplifies spectral interpretation by relating systematic spectral variations to oxide composition and fabrication conditions [13]. This approach groups spectra according to dominant compositional and processing-related variations, including contributions from mixed or poorly crystalline oxide phases that may not correspond to a single library spectrum. The resulting principal-component groupings provide both phase-identification information and insight into the fabrication conditions associated with each oxide composition, making the workflow particularly useful for non-specialist users seeking to adjust fabrication parameters to achieve a desired oxide composition.

In this work, VO_2_, V_2_O_5_, V_3_O_7_, and V_6_O_13_ thin films were produced using a modified sol–gel process [14]. Two samples produced by this fabrication procedure were assessed via GI-XRD to determine whether the diffraction data were consistent with the intended crystalline phases. Power excitation was controlled to avoid samples damage that would alter the characterization. After verifying V_2_O_5_ and VO_2_ phase formation, a detailed power-analysis study was conducted to determine the optimal laser power density for obtaining strong Raman scattering without damaging the samples. Coupling Raman spectroscopy with PCA provided an effective approach for identifying stoichiometric trends within the VOx thin films. Specifically, Raman measurements were used to correlate stoichiometry with fabrication parameters, including chamber pressure, temperature, and curing time. 

Because Raman analysis can be performed quickly and nondestructively, accurate PCA modeling enables a data-driven feedback loop that translates complex spectral data into actionable fabrication guidance for non-specialist users. The model was also evaluated using thermally aged vanadium samples, extending its application beyond synthetic thin films to naturally formed oxides. By gathering larger data sets, the approach will offer the opportunity to create a database that will help identify oxides and fabrication processes for desired outcomes to be further augmented in the future by using more sophisticated machine learning and artificially intelligent tools [15].

## 2. Materials and Methods

In a typical procedure for fabricating thin-film standards, a vanadium alkoxide spin-coating solution was synthesized by mixing 0.658 g of VOCl_3_ (99%, 3.8 mmol) and 1.756 g of (±)-epichlorohydrin (purum, >99%, 18.9 mmol) in 2 mL of ethanol (200 proof, anhydrous) and allowing the mixture to react overnight at room temperature (orange to yellow) [16]. This solution was spin-coated onto clean 1-inch quartz or Si substrates (1500 rpm for 30 s, then 4500 rpm for 60 s). Reaction of the alkoxide with ambient moisture induces hydrolysis and the formation of a complex mixture of hydrated VOxs and alkoxides. This film was cured by heating for 15 min at 150 °C in air, after which additional layers were spin-coated, if desired. To create thinner films, the spin-coating solution was serially diluted by a factor of two with additional ethanol. All reagents were used as received from Sigma-Aldrich (St. Louis, MO, USA).

To convert this complex VOx film to the desired oxide, it was treated under a prescribed atmosphere and temperature. V_2_O_5_ films were synthesized by treatment in air at 200 °C for up to one week, depending on thickness. VO_2_ films were synthesized by treatment under rough vacuum (~10^−3^ Torr) at 500 ± 5 °C (heating rate of 10 °C min^−1^) for up to 4 h and were cooled naturally before removal from vacuum.

To corroborate initial results, GI-XRD measurements were performed using a Bruker (Billerica, MA, USA) D8 Advance diffractometer equipped with a Cu Kα radiation source (λ = 1.5418 Å) and a LynxEye (Stockholm, Sweden) position-sensitive detector. Thin film samples were prepared by mounting them on standard sample holders, ensuring that the surface was clean, flat, and free of contaminants. The sample alignment was verified using the instrument’s alignment tools to minimize tilt and misorientation.

Raman spectroscopy measurements were performed using a Renishaw (Gloucestershire, UK) inVia™ Qontor™ microscope system. The microscope system operates in reflection mode, provides multiple wavelength channels, and uses a laser source with output powers of up to 100 mW and a built-in spectrometer. This configuration enables precise laser focusing on the samples and maximizes the signal-to-noise ratio. Raman spectroscopy was conducted on the vanadium samples using the confocal Raman microscope with a 785 nm laser source. The instrument provides a tunable laser-power range from 2.1 µW (120 W cm^−2^) to ~25 mW (1.4 MW cm^−2^). At 50× magnification, the spot size was measured to be approximately 1.5 µm in diameter. The configuration used for all measurements was a laser exposure time of 30 s. 

The acquired spectral data consisted of Raman intensity values recorded as a function of wavenumber, expressed in cm^−1^. The wavenumber range was limited to 100–1100 cm^−1^ with 1 cm^−1^ resolution. The raw spectral data were transferred to MATLAB R2022b (Natick, MA, USA) as numerical datasets, with each spectrum represented by a vector of intensity values associated with the corresponding wavenumber axis. The datasets were analyzed using the PLS_Toolbox extension developed by Eigenvector Research, Inc. Each Raman dataset was assigned an independent class label on a per-sample basis, unless otherwise specified. The spectra were preprocessed using standard normal variate normalization, mean centering, and Savitzky–Golay smoothing with a window width of 15 cm^−1^. The preprocessed data were then transformed into principal-component space. The resulting principal-component scores represent the coordinates of each spectrum along orthogonal directions that capture progressively decreasing amounts of variance in the dataset. Plotting the scores for PC1, PC2, and PC3 in two- or three-dimensional space enables samples with similar spectral profiles to cluster spatially. A two-dimensional model was used as default and expanded to three dimensions if more than two oxide species are expected.

A set of thermally aged vanadium samples was evaluated in addition to the synthetic thin-film standards. Stock pieces of vanadium were procured from Sigma-Aldrich (St. Louis, MO, USA), trimmed into rectangular coupons approximately 24 mm × 16 mm × 4 mm, and thermally treated on a hotplate.

## 3. Results and Discussion

**Figure 1:**
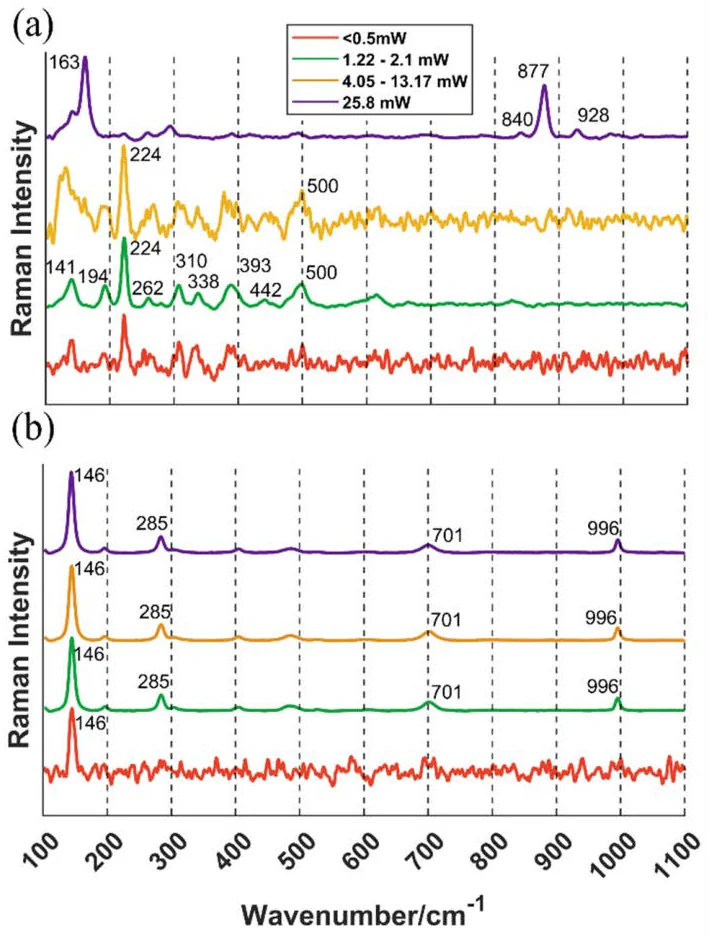
Raman spectra of the VO_2_ thin film (**a**) and the V_2_O_5_ thin film (**b**) measured at laser power ranging from 2.1 µW to 25.8 mW. Each spectrum shown in the figure is an average of its respective power range shown in the legend.

One of the key motivations for using Raman spectroscopy for sample analysis is the inherent nondestructive nature of the technique. However, because VOx materials are extremely sensitive to environmental conditions, including temperature and relative humidity, the measurement conditions must be carefully controlled to avoid unwanted changes to the sample surface. Previous studies using Raman spectroscopy for VOx characterization have cautioned that thermally induced sample oxidation or other surface damage may occur when the laser power density is too high [17,18,19]. However, the exact safe operating threshold depends on many parameters, including laser wavelength, spot size, power, exposure time, and the properties of the sample being analyzed. To study this effect in the present system, two thin films were analyzed using multiple Raman laser-power settings. Prior to Raman analysis, the two samples underwent GI-XRD analysis, shown in Figure S1, which confirmed the synthesis of the intended stoichiometric phases, V_2_O_5_ and VO_2_ [20,21,22]. The two samples were measured in a grid-like pattern at nine different laser powers ranging from 2.1 µW to 25.8 mW. The resulting Raman spectra, shown in Figure 1, were separated into four laser-power regions based on chemical changes observed in the VO_2_ sample’s Raman spectrum (Figure 1a).

The red spectra shown in Figure 1 were measured below 0.5 mW and exhibited noisy and unrecognizable Raman fingerprints for both samples. This region was determined to provide insufficient laser power to excite observable Raman scattering in the thin-film samples. The green spectra, measured between 1.22 mW and 2.1 mW, were representative of VO_2_(M1) with peaks at 140, 194, 224, 262, 310, 338, 393, 442, and 500 cm^−1^, and α-V_2_O_5_ with peaks at 146, 285, 701, and 996 cm^−1^, as verified against the literature [17,18,19]. This power range was determined to be the acceptable operating range because both Raman fingerprints matched reported spectra. The third group, shown in yellow, was measured between 4.05 mW and 13.17 mW and showed deviations from the original spectra measured in the green group for VO_2_ only. While the Raman peaks were different, a VO_2_ spectrum was still observable, including the peaks at 224 cm^−1^ and 500 cm^−1^. These changes are consistent with laser-induced partial phase transformation. Finally, the fourth group, shown in purple, was measured at 25.8 mW, the maximum output of the Raman instrument. In this region, the VO_2_ sample no longer resembled the original spectrum, and new peaks were present. These results indicate that the applied laser power was sufficient to irreversibly alter the sample surface. Interestingly, the V_2_O_5_ sample was resilient to all applied laser powers. This is consistent with the material properties of each respective VOx, as VO_2_ is known to have a temperature-induced metal-insulator transition, whereas V_2_O_5_ is thermally more stable and synthetically reproducible [23,24].

**Figure 2:**
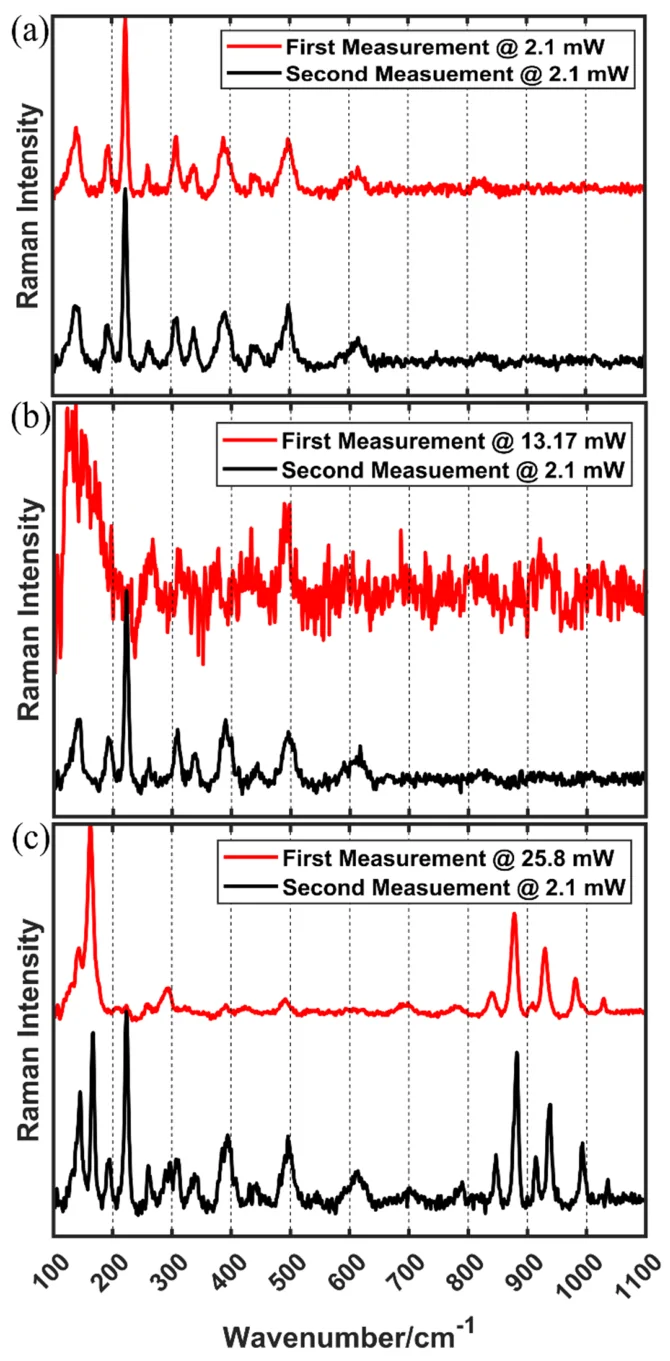
Raman Spectra of VO_2_(M1) acquired at 2.1 (**a**), 13.17 (**b**), and 25.8 (**c**) mW, illustrating repeatability, reversible spectral changes, and irreversible sample alteration, respectively.

An additional study was conducted on the VO_2_ sample to determine if the observed change in the Raman fingerprint was permanent. For this study, two successive Raman spectra were acquired at a single point on the sample at two different laser powers. Three trials were conducted to individually assess the three noticeable phase transitions from Figure 1a. The first trial acquired both spectra at 2.1 mW (Figure 2a). This confirms that measurements within the tolerable operating range reliably produce a VO_2_(M1) Raman spectrum. The second trial measured a spot on the sample first at 13.17 mW, followed by a second measurement at 2.1 mW (Figure 2b). The first measurement induced the spectral changes observed in Figure 1, while the second measurement assessed whether this transition was permanent. Since the second measurement at 2.1 mW matches the original spectrum from Figure 2a, the intermediate laser-power range was determined not to cause permanent sample damage. The third trial challenged the sample with 25.8 mW of laser power, which induced an irreversible change in the VO_2_ sample. Reassessing this location at the tolerable laser power of 2.1 mW showed new Raman peaks between 800 cm^−1^ and 1000 cm^−1^.

Our results suggest that an upper power limit should be restricted when measuring thin-film samples to avoid undesired phase transitions, even if they are temporary. Since both samples produce the expected Raman spectra for the target composition between 1.22 mW and 2.1 mW, this range was selected as the laser-power operating regime. Furthermore, our results indicate that visual inspection alone is not sufficient to determine if laser-induced damage has occurred, as the transition at the lower power was not visible using the optical microscope. This step is important and recommended for guidance in the proper parameter selection to pursue the correct analysis.

Next, a group of 25 thin-film samples was fabricated using the previously described technique with pyrolysis temperatures ranging from 400 °C to 500 °C, pressures ranging from atmospheric pressure to rough vacuum (10^−3^ mbar) and high vacuum (10^−5^ mbar), epoxide stoichiometries of either 3.5 or 5, and pyrolysis durations ranging from 24 min to 240 min. The VOx Ellingham diagram, published by Brahlek et al., shows that fabrication of VO_2_ can be difficult due to the required balance of partial oxygen pressure and annealing temperature [25]. The goal was to use Raman spectroscopy to identify a fabrication recipe that yields consistent results for synthesizing pure VO_2_ films. Raman spectroscopy measurements were acquired at nine locations for each sample, averaged on a per-sample basis, and imported into MATLAB R2022b for PCA.

**Figure 3:**
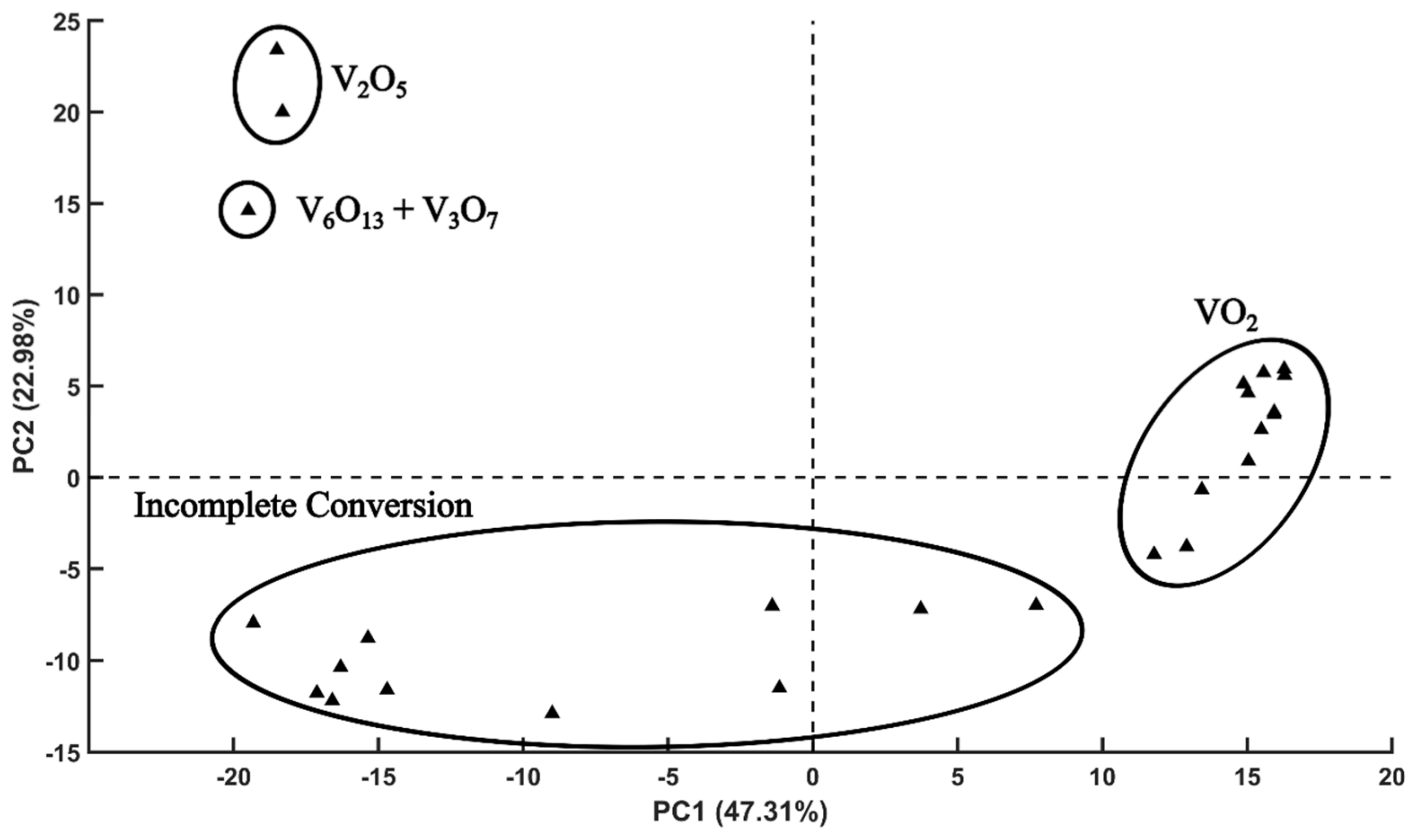
Principal component score plot of 25 VOx thin films, showing clusters associated with distinct Raman spectral characteristics and fabrication conditions.

In general, PCA reduces the dimensionality of a complex dataset into orthogonal principal components (principal component 1, 2, etc.) ordered by the amount of variance captured. When applied to a dataset of Raman spectra, the principal component scores and loadings capture the spectral variation that distinguishes a given sample from others within the dataset. Applying PCA to VOx samples with unknown stoichiometries provides a simplified method for grouping spectrally similar samples into clusters. The resulting 2D PC2 versus PC1 plot for this dataset of 25 thin films is shown in Figure 3. The VO_2_ and V_2_O_5_ reference samples, previously confirmed by GI-XRD, were also included in the analysis to help classify the sample groupings in the principal-component domain. Eleven samples had near-featureless Raman spectra, suggesting that the chemical precursor had not fully converted to a stable VOx phase. These samples were grouped together and classified as “Incomplete Conversion.” Interestingly, the Raman spectra from this group, shown in Figure S2, all contained a peak at 500 cm^−1^, and three of the samples also contained a peak at 224 cm^−1^, suggesting partial conversion to VO_2_.

All remaining samples with a positive PC1 score featured VO_2_ Raman spectra. Three samples projected strongly in the positive PC2 direction with negative PC1 scores. Upon comparison with literature, one sample matched the α-V_2_O_5_ reference sample, whereas another exhibited Raman features consistent with a V_3_O_7_ and V_6_O_13_ mixed oxide [18,19,23]. Specifically, a triplet of peaks at 840, 880, and 930 cm^−1^ was consistent with V_3_O_7_, while additional strong Raman bands at 129, 422, and 501 cm^−1^ corresponded to V_6_O_13_. The cumulative variance captured by the two-dimensional PCA model was 70.29%. This metric could be increased if the samples within the “Incomplete Conversion” group were excluded. However, these results suggest that including this spectral group produces distinguishable clusters that separate samples by their stoichiometry.

For further sample inspection, the VO_2_ films were analyzed to determine their thickness and to evaluate whether a sample’s Raman fingerprint can be used to estimate film thickness. VO_2_ samples were fabricated at various thicknesses on silicon (Si) substrates, which feature a strong Raman peak at 520 cm^−1^ [26]. A thin film deposited on a substrate attenuates the Raman signal from the underlying layer, and the degree of attenuation can be correlated to the total film thickness [25]. Various thicknesses were achieved by diluting the chemical precursor, producing films approximately one-quarter to twice the typical thickness used in this study. Three Raman spectra were acquired from each fabricated sample and averaged. The ratio between the mean intensity of the main VO_2_ peak at 224 cm^−1^ and the Si peak at 520 cm^−1^ for each sample was plotted as a function of sample thickness, as shown in Figure S3. The VO_2_ thin-film samples shown in Figure S3 feature a darker color than the films on glass due to Si substrate reflection. To measure the thickness of each thin-film sample, ellipsometry data were acquired on each sample and modeled using the CompleteEase 6.54 software package (J.A. Woollam, Lincoln, NE, USA), as shown in Figure S3. SEM images of FIB lift-outs were used to validate the ellipsometry model and the fitted optical constants (n = 3.120 and k = 0.340). Our results indicate successful fabrication of VOx thin films with thicknesses ranging from 22.7 nm to 182.1 nm, which correlated with normalized Raman intensity.

The final composition of the V_3_O_7_ + V_6_O_13_ mixed film from Figure 3 was found to depend on the absolute film thicknesses and the thickness ratio of the two films. Samples containing high absolute thicknesses, created using multiple V_2_O_5_ layers, exhibited a significant number of visible inhomogeneities. With a well-defined fabrication protocol for both V_2_O_5_ and VO_2_, an additional set of intervalent VOx standards was produced using this process. Ten total samples were fabricated for this study and are summarized in Table S1. Four samples were replicates of the previously defined VO_2_ recipe, pyrolyzed in a rough vacuum at 500 °C for 240 min on bare silica with no V_2_O_5_ layer, and served as controls in the dataset. One of these four control samples had an increased pyrolysis time of 480 min. The remaining six samples used the standard VO_2_ recipe on top of one, two, or three layers of V_2_O_5_ thin film deposited on a silica substrate. Pyrolysis times of 240 min and 480 min, with three samples at each duration, were used for the top VO_2_ layer.

**Figure 4:**
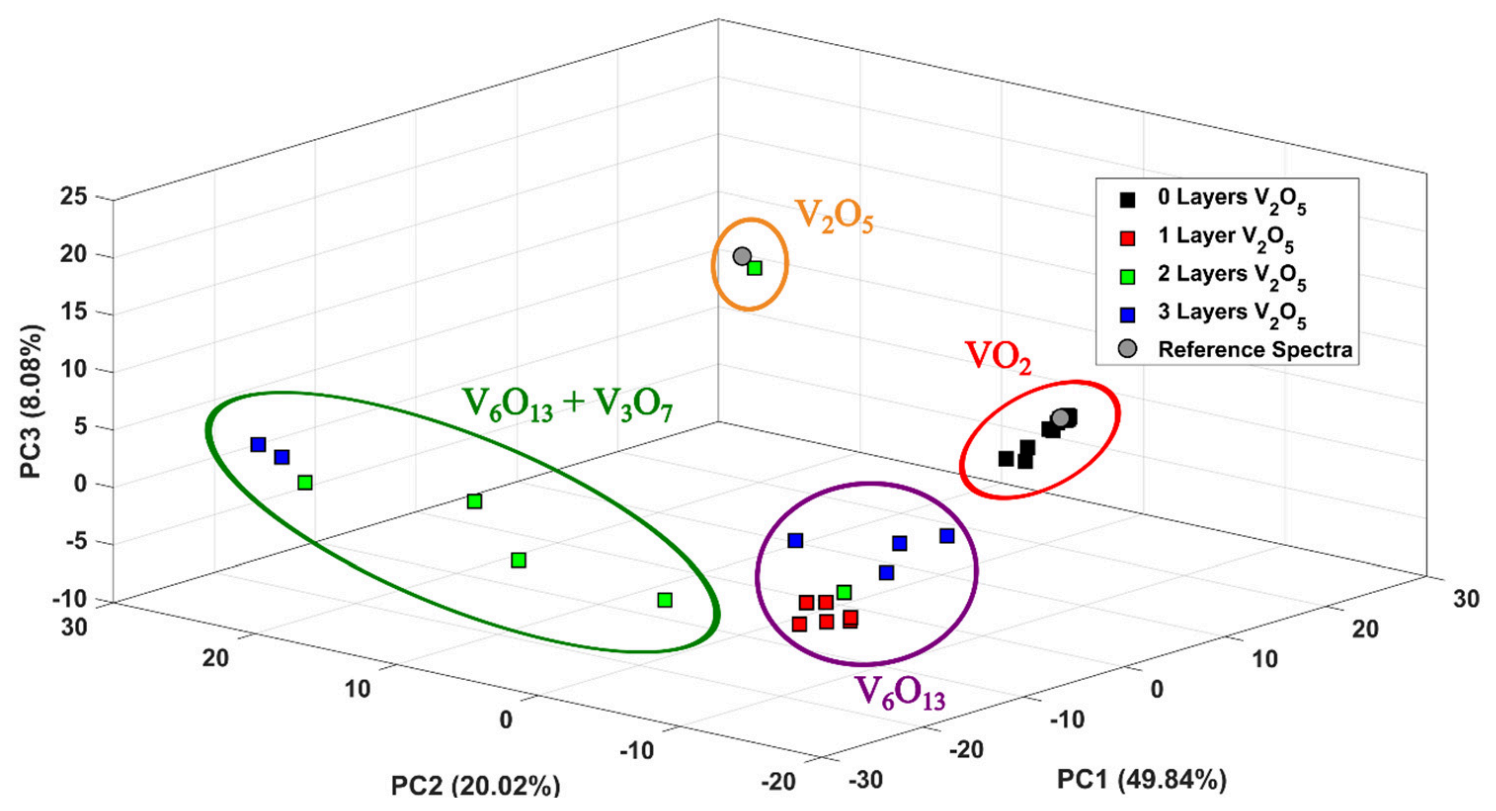
Three-dimensional principal-component score plot of Raman spectra from VO_2_ thin films deposited on silica with 0–3 layers of V_2_O_5_, showing clusters associated with different VOx phases.

Raman spectroscopy measurements were acquired at three locations for each sample. The gathered spectra were not averaged due to color variation observed across the sample face, which was expected to represent spatial variation in oxide composition, as shown in Figure S4. Therefore, each sample had three spectra included in the PCA model applied to the dataset. A three-dimensional PCA was applied to the dataset to account for the increased complexity associated with the additional oxide phases. The PC3 vs. PC2 vs. PC1 scores plot shown in Figure 4 groups the spectra into four categories. First, there were 13 spectra that scored along the positive PC1 axis without projecting in either the positive or negative direction of the other principal components. These scores included the three spectra acquired from each of the four VO_2_ samples with zero layers of V_2_O_5_, plus one spectrum from the VO_2_ reference sample. The remaining samples scored in the negative PC1 direction. Of these remaining samples, two scored strongly in the positive PC3 direction: one spectrum from a VO_2_ film deposited on two layers of V_2_O_5_, plus the spectrum from the V_2_O_5_ reference sample. 

The remaining 17 spectra were separated into two primary groups according to their PC2 score. The samples with a negative PC2 score match a V_6_O_13_ Raman fingerprint with major peaks located at 129, 153, 204, 298, 340, 422, and 501 cm^−1^. The remaining samples with a positive PC2 score formed a group of six spectra. These spectra share many of the same Raman peaks as the V_6_O_13_ group. However, analysis of the corresponding biplot, Figure S5a, shows that the distinguishing features between the two groups are strong Raman peaks at 167 cm^−1^ and 880 cm^−1^, which correlate with V_3_O_7_, as shown in Figure S5d. Combining the characteristic biplot with the sample principal-component scores gives insight into the meaning of each principal component. For example, in this model, PC1 can be viewed as a binary operator to determine whether a sample contains VO_2_. Similarly, PC3 denotes whether V_2_O_5_ is present, and the strength of the positive PC3 score is correlated with the abundance of V_3_O_7_. Finally, these results demonstrate that the recipe for VO_2_ on bare silica can reliably reproduce a stable oxide. Furthermore, all VO_2_ films deposited on a single layer of V_2_O_5_ produced a V_6_O_13_ thin film.

Previous reports indicate that V_6_O_13_ produces a very weak Raman spectrum, such that its spectral response may be obscured when other oxides are present [18]. Producing a V_6_O_13_ spectrum without spectral interference therefore indicates a pure oxide, demonstrating that pristine V_6_O_13_ thin-film standards can be produced. As expected, increasing the amount of the V(V) component raises the average oxidation state from 4.33 for V_6_O_13_ to 4.67 for V_3_O_7_. However, given that each film should contain approximately the same amount of V, a single layer of VO_2_ on a single layer of V_2_O_5_ should be insufficient to form V_6_O_13_. Residual organic species in the deposited layers likely play a role as redox-active components in the final composition. This may explain the deviation in synthesis parameters from the Ellingham diagram and is supported by the fact that increasing the pyrolysis time did not appear to affect the final composition and that V_2_O_5_ films could not be converted to VO_2_ films by pyrolysis under vacuum alone [25,27].

In addition to spin-cast sol–gel oxide standards, a set of oxides were thermally grown on vanadium metal coupons under various conditions and assessed via Raman spectroscopy at 2.1 mW to expand the reference library and provide insight into oxidation-dependent parameters. Ten vanadium coupons were thermally aged according to the protocol shown in Table S2. The final thermally aged VOx samples featured a range of colors on the top surface. While color change is an indicator of oxide growth, the specific color is not always an unambiguous indicator of oxidation state, but rather reflects a combination of total thickness and oxygen content [28,29]. It is therefore imperative to categorize each sample by its chemical composition rather than its physical appearance. Each sample was measured by Raman spectroscopy at nine different locations on the sample surface. The nine spectra were averaged and imported into MATLAB R2022b for further analysis. The three-dimensional PCA model developed for studying intervalent oxides (Figure 4) was applied to the dataset of thermally aged vanadium samples.

**Figure 5:**
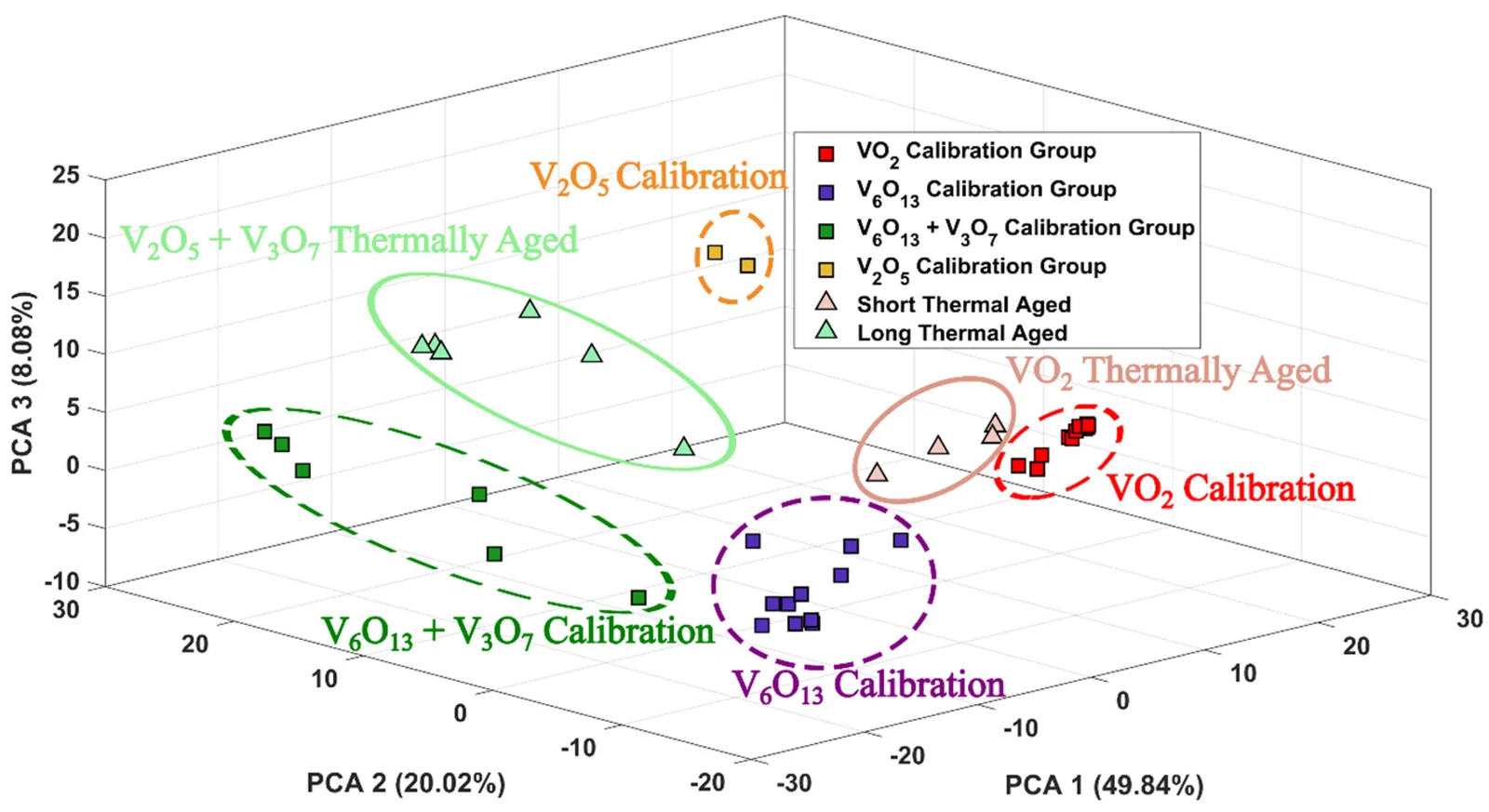
The PCA model developed for intervalent VOxs was applied to the Raman data for 10 thermally aged vanadium samples.

The resulting three-dimensional PCA plot is shown in Figure 5, where each sample’s average Raman spectrum is plotted as a function of its PC3, PC2, and PC1 scores. Markers that represent the principal-component scores from the original model are included for visualization purposes and are color-coded to correspond with the primary groupings in Figure 4. The thermally aged samples were split into two groups corresponding to the polarity of their PC1 scores. Interestingly, organizing the data in this fashion was directly related to the sample aging protocol. Positive PC1 scores were associated with high-temperature (≥300 °C), short-duration (≤4.25 h) treatments, whereas negative PC1 scores were associated with longer exposures at the same temperature (>4.25 h). The model applied to this dataset identifies PC1 as an indicator of VO_2_ growth. Therefore, the data suggest that thermally aged vanadium initially oxidizes predominantly to VO_2_. The samples aged for longer than 4.25 h at high temperatures exhibited complex Raman spectra. The group’s placement on the PCA plot showed positive PC3 scores for all samples, indicating the presence of V_2_O_5_, and positive PC2 scores for all but one sample, indicating the presence of V_3_O_7_. The raw Raman spectra for each sample, sorted by these groupings, are shown in Figure S6. Further analysis of this group shows a strong correlation to the main α-V_2_O_5_ peaks at 146, 285, 701, and 996 cm^−1^, but with an additional triplet of peaks at 840, 880, and 930 cm^−1^, confirming the presence of V_3_O_7_ [17,26].

**Figure 6:**
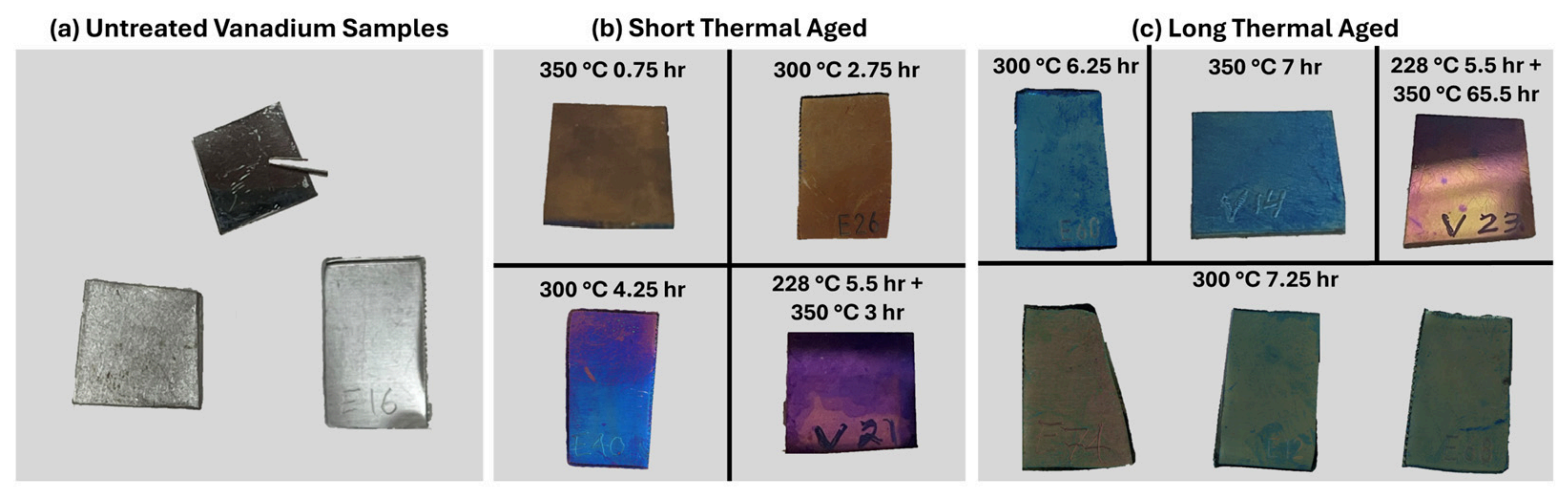
Images of thermally aged vanadium samples, grouped according to their principal-component classifications and thermal-treatment conditions.

The purpose of the study of the thermally aged vanadium samples is two-fold. First, inducing thermally accelerated aging provides a dataset that demonstrates how vanadium oxidizes under controlled conditions. Second, the Raman measurements from this dataset verify the measurement system, expand the reference library for characterization of thin-film standards, and support future rapid assessment of oxidation state. When fabricating chemically synthesized standards, it is important to replicate real-world oxidation phenomena, including naturally occurring oxidation states and thicknesses. The PCA grouping shown in Figure 5 not only demonstrates stoichiometric separation among the samples but also correlates with the duration of thermal treatment. Images of the samples, the thermal treatment recipe, and their corresponding PCA group are shown in Figure 6. By grouping the samples according to their PCA scores, it is concluded that the top layer of natural oxide growth starts as VO_2_ and then transitions to V_2_O_5_, which can rapidly produce other higher-order oxidation states depending on the storage conditions. Additionally, the color patterns of the VOx films follow a similar pattern to that reported for TiO_2_, where the color change cycles from amber to indigo, to blue, to yellow-blue, and back to indigo [30].

## 4. Conclusions

This work demonstrates a data-driven Raman spectroscopy workflow for the rapid, nondestructive characterization of VOx thin films on silica and silicon substrates. By coupling Raman measurements with principal component analysis (PCA), complex spectral data were transformed into interpretable sample groupings that differentiated VO_2_, V_2_O_5_, V_3_O_7_, and V_6_O_13_ and informed the fabrication conditions required to produce each phase. The resulting thin-film standards, summarized in Table 1, provided a reference dataset for PCA model development and enabled classification of naturally and thermally aged vanadium samples. The assignments of V_3_O_7_, V_6_O_13_, and mixed vanadium oxide compositions were supported by comparison with literature-reported Raman-active modes and should be interpreted as predominant compositions rather than identifications of pure phases. Overlapping Raman bands, amorphous contributions, and thickness-dependent spectral effects may also influence the observed groupings.

This approach establishes a foundation for a data-driven characterization framework that converts rapid optical measurements into qualitative, actionable feedback on oxide stoichiometry, processing conditions, and potential sample changes. Rather than relying exclusively on expert spectral interpretation, the PCA model provides a scalable framework for exploratory, data-driven decision support during VOx fabrication and oxidation monitoring. This work did not include formal classification metrics, cross-validation, confidence intervals, or evaluation with an independent test set. Accordingly, the PCA groupings should be viewed as exploratory rather than as a fully validated classification model.

More broadly, this work highlights the value of nondestructive Raman fingerprinting combined with multivariate analysis for adaptive characterization of chemically complex material systems. The PCA groupings provide useful qualitative insight into relationships among Raman spectral features, oxide composition, and processing conditions. Future work may build on these results by evaluating the workflow with larger datasets, replicate measurements, and independently prepared or blind samples to further assess its robustness and broader applicability. Additional complementary characterization, including expanded GI-XRD measurements and techniques such as XPS or TEM, would also help further refine and validate the phase assignments, particularly for V_3_O_7_, V_6_O_13_, and mixed-phase compositions. Additionally, this work can be expanded by implementing more sophisticated artificial intelligence tools to augment the multivariate analysis.

## Figures and Tables

**Table 1:** Summary of the four thin film compositions of VOx that were fabricated. The measured Raman peaks are displayed with major peaks bolded. Sequential recipes are outlined by step numbers.

VOx	Pyrolysis Pressure	Pyrolysis Temperature	Pyrolysis Time	Raman Peaks [cm^−1^]	Image of Film on Silica
V_2_O_5_	Air	400 °C	24–240 min	**146,** 196, 285, 302, 405, 483, 529, 605, **701**, **996**	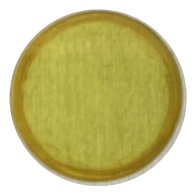
VO_2_ (M1)	Rough Vacuum	500 °C	24–240 min	140, 194, **224**, 262, 310, 338, 393, 442, **500**	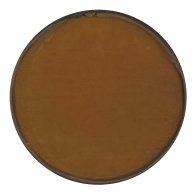
V_6_O_13_	(1) Air (2) Rough Vacuum	(1) 400 °C (2) 500 °C	(1) 24 min (2) 240 min	**129**, 153, 204, 298, 340, **422, 501**	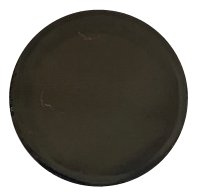
V_3_O_7_ + V_6_O_13_	(1) Air (2) Rough Vacuum (3) Rough Vacuum	(1) 400 °C (2) 500 °C (3) 500 °C	(1) 24 min (2) 240 min (3) 240 min	**129, 167,** 295, 340, 427, 501, **846, 882,** 915, **936**, 991, 1033	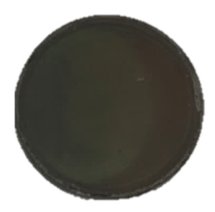

## Data Availability

The data supporting the findings of this study are available from the corresponding author upon reasonable request, subject to privacy restrictions.

## Supplementary Materials

The following supporting information can be downloaded at https://www.mdpi.com/article/10.3390/s26196308/s1: Figure S1: Above displays the GI-XRD spectra of the two thin film samples used for the laser intensity study, fit to known phases from the ICDD PDF 5+ database using GSAS II [20,21]. The analysis confirmed the V_2_O_5_ and VO_2_ standards developed and used in the study were the intended phases synthesized. The V_2_O_5_ standard was fit to ICDD 00-041-1426, experimentally determined via Shultz et al 1988 [21]. The VO_2_ standard was fit to ICDD 00-009-0142, experimentally determined by Andersson et al 1954 [22]; Figure S2: Raman spectra from each sample in the dataset from Figure 3. The spectra are grouped together in the same primary groups in the PC2 versus PC1 plot: VO_2_ (a), Incomplete Conversion (b), V_2_O_5_ (c), V_3_O_7_ (d); Figure S3: (a) Mean square error obtained from ellipsometry model for each thickness. (b) The Ramn intensity peak for VO_2_ (224 cm^−1^) normalized to the Raman peak for Silicon (520 cm^−1^) shows an attenuation that correlates with film thickness. (c) Ellipsometry model of the 1-layer sample which is 99.3 nm. Plot shows the model accurately fits the raw ellipsometry data. (d) Corresponding n and k values, optical constants, for the 1-layer sample; Figure S4: Optical images of the VO_2_ on Si samples used for the VO_2_ on V_2_O_5_ study sorted by the total number of V_2_O_5_ layers on the sample before adding the VO_2_ layer on top. This range includes 0 layers (a), one layer (b), two layers (c), and three layers (d); Figure S5: The biplot corresponds to the PCA plot shown in Figure 5. (a) The biplot was filtered to show only the most influential variables (Raman peaks) in the PC1 and PC2 direction. The Raman spectra of the samples sorted by the three primary PCA groups are shown: VO_2_ (b), V_6_O_13_ (c), and V_6_O_13_ + V_3_O_7_ (d). The asterisks in (d) denote influential peaks at 880 cm^−1^ and 930 cm^−1^ driving the group along the positive PC2 axis; Figure S6: Raman spectra of the thermally treated Vanadium samples. The short aged samples (a) feature a VO_2_ spectra (with the VO_2_ reference shown first) with V_3_O_7_ present in some samples and the long aged samples (b) are mostly a mix of V_2_O_5_ and V_3_O_7_; Table S1: Fabrication recipes for the 10 thin films used to study thin film intervalent oxide production. Each sample was measured via Raman spectroscopy in three different locations; Table S2: Thermal treatment recipes for the 10 vanadium samples.

## References

[1] Baddour-Hadjean R., Pereira-Ramos J.P. (2010). Raman microspectrometry applied to the study of electrode materials for lithium batteries. Chem. Rev..

[2] Tiwari P., Das K., Katare R. (2024). UV and FTIR Studies of Pure PMMA and V_2_O_5_ Doped Films. Int. J. Creat. Res. Thoughts.

[3] Liang J., Hou L., Li J. (2016). Frequency tunable perfect absorber in visible and near-infrared regimes based on VO_2 phase transition using planar layered thin films. J. Opt. Soc. Am. B.

[4] Jiang C., He L., Xuan Q., Liao Y., Dai J.G., Lei D. (2024). Phase-change VO(2)-based thermochromic smart windows. Light. Sci. Appl..

[5] Jerominek H. (1993). Vanadium oxide films for optical switching and detection. Opt. Eng..

[6] Hu P., Hu P., Vu T.D., Li M., Wang S., Ke Y., Zeng X., Mai L., Long Y. (2023). Vanadium Oxide: Phase Diagrams, Structures, Synthesis, and Applications. Chem. Rev..

[7] Choi J.H., Lee H.T., Kim J., Park M., Sun S., Kim G.H., Kwon M.H., Park H.R., Sohn C. (2026). AI-Optimized Vanadium Oxide Multilayers for More Than 20-fold Enhancement in Bolometric Performance. Adv. Sci..

[8] Surnev S., Ramsey M.G., Netzer F.P. (2003). Vanadium oxide surface studies. Prog. Surf. Sci..

[9] Wachs I.E. (2013). Catalysis science of supported vanadium oxide catalysts. Dalton Trans..

[10] Dai H. (2015). Environmental catalysis: A solution for the removal of atmospheric pollutants. Sci. Bull..

[11] Davydov D.A., Rempel A.A. (2009). Refinement of the V-O phase diagram in the range 25–50 at % oxygen. Inorg. Mater..

[12] Cao Z., Li S., Xie W., Du G., Qiao Z. (2015). Critical evaluation and thermodynamic optimization of the V–O system. Calphad.

[13] Adar F. (2021). A Simple Introduction to Raman Spectral Identification of Organic Materials. Spectroscopy.

[14] Guzman G., Morineau R., Livage J. (1994). Synthesis of vanadium dioxide thin films from vanadium alkoxides. Mater. Res. Bull..

[15] Smith R., Spano T.L., McDonnell M., Drane L., Gibbs I., Miskowiec A., Niedziela J.L., Shields A.E. (2025). Interpretable machine learning models classify minerals via spectroscopy. Sci. Rep..

[16] Leventis T.M.F.N., Sotiriou-Leventis C., Winiarz J.G. (2015). Nanoporous Vanadium Oxide Aerogels and Methods of Preparation. https://patents.google.com/patent/US20150284258A1/en.

[17] Shvets P., Dikaya O., Maksimova K., Goikhman A. (2019). A review of Raman spectroscopy of vanadium oxides. J. Raman Spectrosc..

[18] Shvets P., Krylov A., Maksimova K., Goikhman (2024). Raman spectroscopy of Wadsley phases of vanadium oxide. J. Raman Spectrosc..

[19] Zhang C., Yang Q., Koughia C., Ye F., Sanayei M., Wen S.-J., Kasap S. (2016). Characterization of vanadium oxide thin films with different stoichiometry using Raman spectroscopy. Thin Solid Films.

[20] Kabekkodu S.N., Dosen A., Blanton T.N. (2024). PDF-5+: A comprehensive Powder Diffraction File™ for materials characterization. Powder Diffr..

[21] Schulz D., Larson F., McCarthy G. (1988). Powder Diffraction Data for V_2_O_5_.

[22] Andersson G., Paju J., Lang W., Berndt W. (1954). Studies on Vanadium Oxides. I. Phase Analysis. Acta Chem. Scand..

[23] Feng D., Zhang J., Li M., Chen M., Zhao B. (2020). Phase Equilibria of the SiO2–V2O5 system. Ceram. Int..

[24] Morin F.J. (1959). Oxides Which Show a Metal-to-Insulator Transition at the Neel Temperature. Phys. Rev. Lett..

[25] Brahlek M., Zhang L., Lapano J., Zhang H.-T., Engel-Herbert R., Shukla N., Datta S., Paik H., Schlom D.G. (2017). Opportunities in vanadium-based strongly correlated electron systems. MRS Commun..

[26] Dzhagan V.M., Valakh M.Y., Isaieva O.F., Yukhymchuk V.O., Stadnik O.A., Gudymenko O.Y., Lytvyn P.M., Kulbachynskyi O.A., Yefanov V.S., Romanyuk B.M. (2024). Raman fingerprints of different vanadium oxides as impurity phases in VO2 films. Opt. Mater..

[27] Brauer G. (1963). Handbook of Preparative Inorganic Chemistry.

[28] Ledinsky M., Paviet-Salomon B., Vetushka A., Geissbühler J., Tomasi A., Despeisse M., De Wolf S., Ballif C., Fejfar A. (2016). Profilometry of thin films on rough substrates by Raman spectroscopy. Sci. Rep..

[29] Zhu M., Zhang D., Jiang S., Liu S., Qi H., Yang Y. (2021). Phase evolution and thermochromism of vanadium oxide thin films grown at low substrate temperatures during magnetron sputtering. Ceram. Int..

[30] Antończak A.J., Stępak B., Kozioł P.E., Abramski K.M. (2013). The influence of process parameters on the laser-induced coloring of titanium. Appl. Phys. A.

