# Supplementary material for: Methods for Rapid and Smart Characterization of Vanadium Oxide Thin Films and Thermally Aged Samples Using Raman Spectroscopy and Multivariate Analysis
Source: Sensors (Basel). 2026 Oct 6;26(19):6308. doi: 10.3390/s26196308 (PMC13667691; doi:10.3390/s26196308)
Supplement: Supplementary file 1 [file sensors-26-06308-s001.zip › sensors-4574236-supplementary.pdf]

Supporting Information

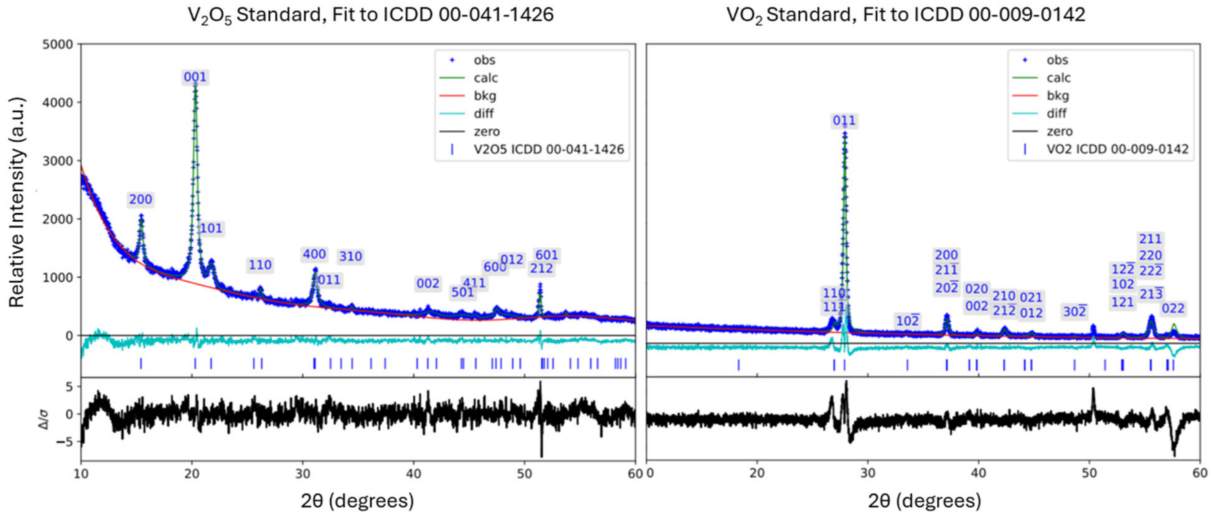

Figure S1. Above displays the GI-XRD spectra of the two thin film samples used for the laser intensity study, fit to known phases from the ICDD PDF 5+ database using GSAS II [20-21]. The analysis confirmed the V_2O_5 and VO_2 standards developed and used in the study were the intended phases synthesized. The V_2O_5 standard was fit to ICDD 00-041-1426, experimentally determined via Shultz et al 1988 [21]. The VO_2 standard was fit to ICDD 00-009-0142, experimentally determined by Andersson et al 1954 [22].

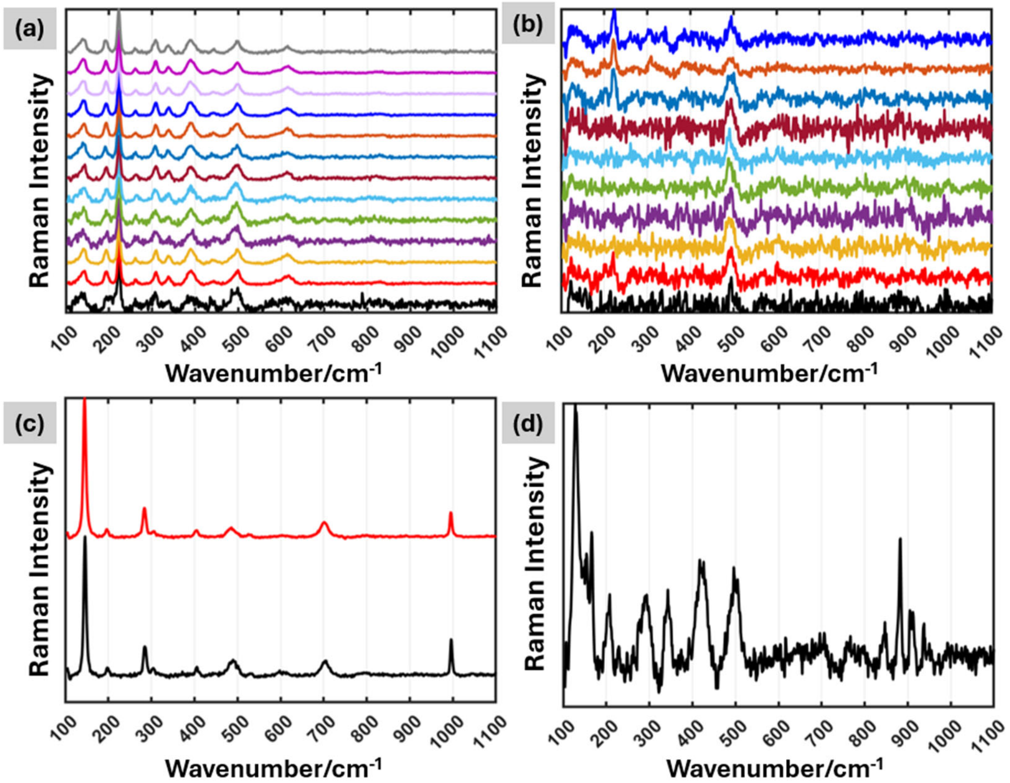

Figure S2. Raman spectra from each sample in the dataset from **Figure 3.** The spectra are grouped together in the same primary groups in the PC2 versus PC1 plot: VO_2 (a), Incomplete Conversion (b), V_2O_5 (c), V_3O_7 (d).

(a)

Sample	Thickness (nm)	Mean Square Error (nm)
2 - Layer	182.1	1.3
1 - Layer	99.3	1.8
½ - Layer	53.1	1.4
¼ - Layer	22.7	0.3

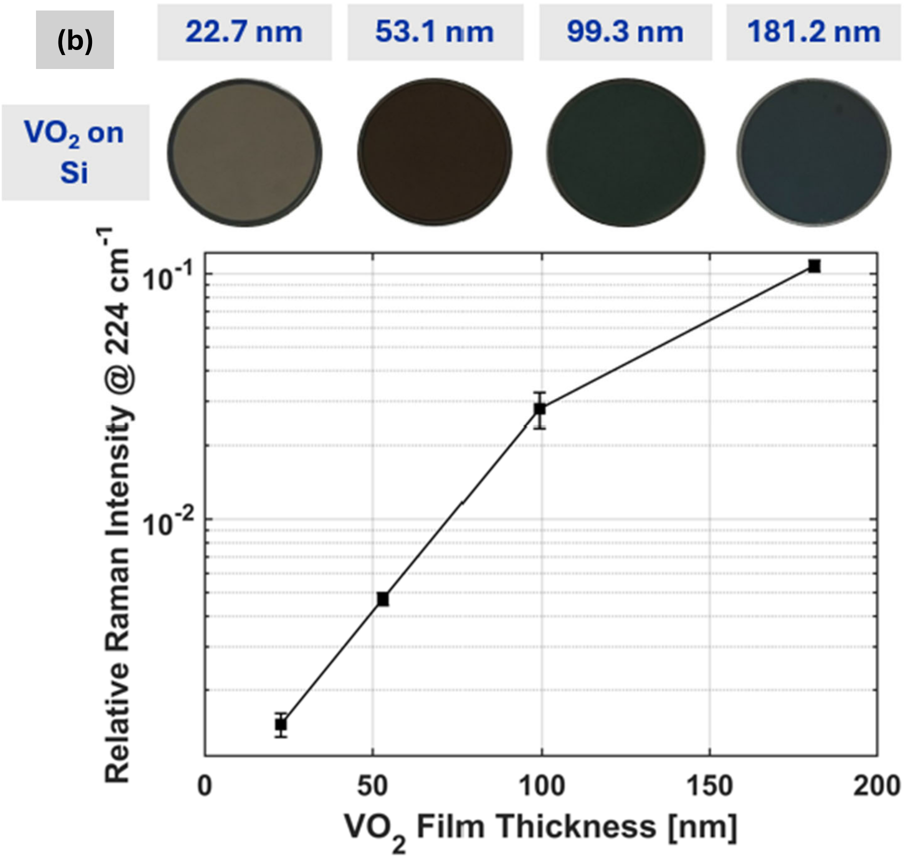

(c)

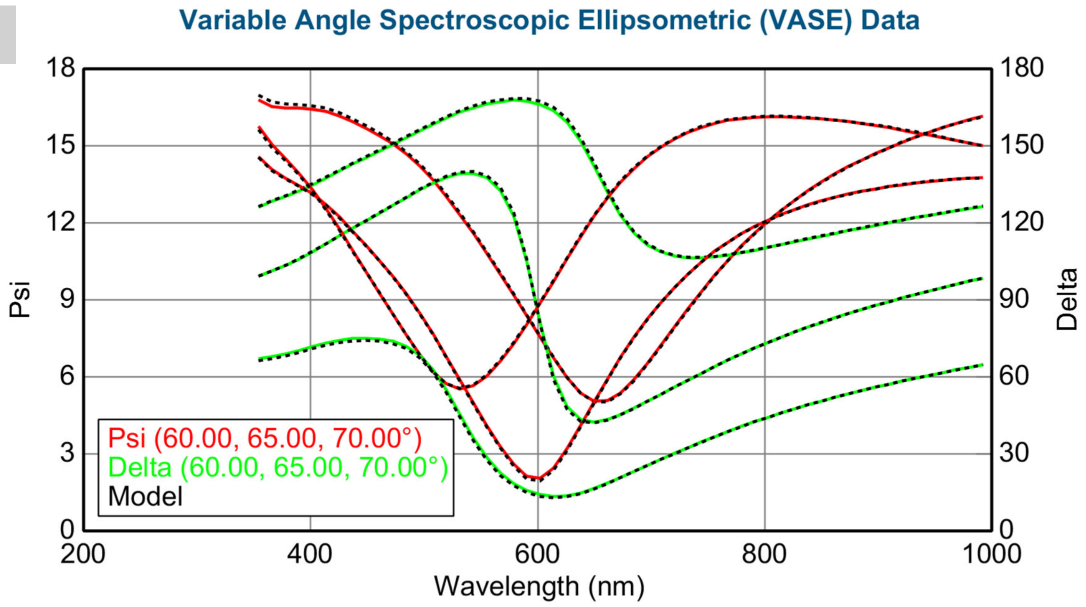

(d)

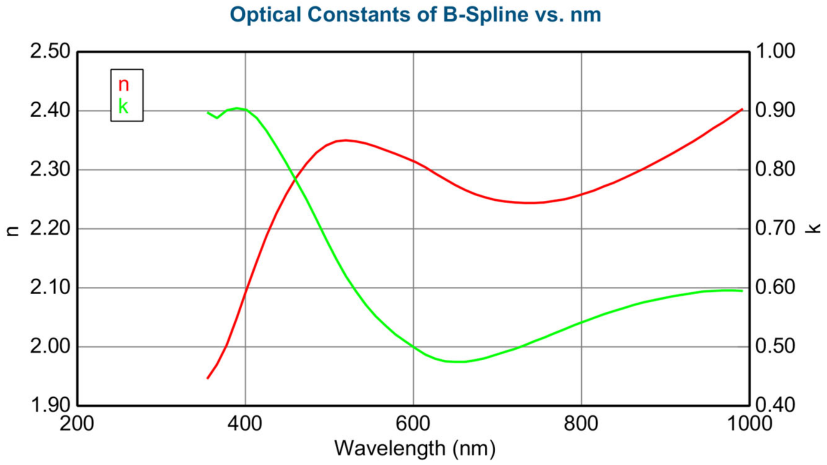

Figure S3. (a) Mean square error obtained from ellipsometry model for each thickness. (b) The Ramn intensity peak for VO_2 (224 cm^{-1}) normalized to the Raman peak for Silicon (520 cm^{-1}) shows an attenuation that correlates with film thickness. (c) Ellipsometry model of the 1-layer sample which is 99.3 nm. Plot shows the model accurately fits the raw ellipsometry data. (d) Corresponding n and k values, optical constants, for the 1-layer sample.

Sample #	# of V_2O_5 base layers	VO_2 Pyrolysis time	Stoichiometry determined by Raman
Sample 1 (Control)	0	240 min	Spectra 1- VO_2<br>Spectra 2- VO_2<br>Spectra 3- VO_2
Sample 2 (Control)	0	240 min	Spectra 1- VO_2<br>Spectra 2- VO_2<br>Spectra 3- VO_2
Sample 3 (Control)	0	240 min	Spectra 1- VO_2<br>Spectra 2- VO_2<br>Spectra 3- VO_2
Sample 4 (Control)	0	480 min	Spectra 1- VO_2<br>Spectra 2- VO_2<br>Spectra 3- VO_2
Sample 5	1	240 min	Spectra 1- V_6O_{13}<br>Spectra 2- V_6O_{13}<br>Spectra 3- V_6O_{13}
Sample 6	1	480 min	Spectra 1- V_6O_{13}<br>Spectra 2- V_6O_{13}<br>Spectra 3- V_6O_{13}
Sample 7	2	240 min	Spectra 1- V_6O_{13} + V_3O_7<br>Spectra 2- V_2O_5<br>Spectra 3- V_6O_{13} + V_3O_7
Sample 8	2	480 min	Spectra 1- V_6O_{13} + V_3O_7<br>Spectra 2- V_6O_{13}<br>Spectra 3- V_6O_{13} + V_3O_7
Sample 9	3	240 min	Spectra 1- V_6O_{13} + V_3O_7<br>Spectra 2- V_6O_{13}<br>Spectra 3- V_6O_{13} + V_3O_7
Sample 10	3	480 min	Spectra 1- V_6O_{13}<br>Spectra 2- V_6O_{13}<br>Spectra 3- V_6O_{13}

Table S1. Fabrication recipes for the 10 thin films used to study thin film intervalent oxide production. Each sample was measured via Raman spectroscopy in three different locations.

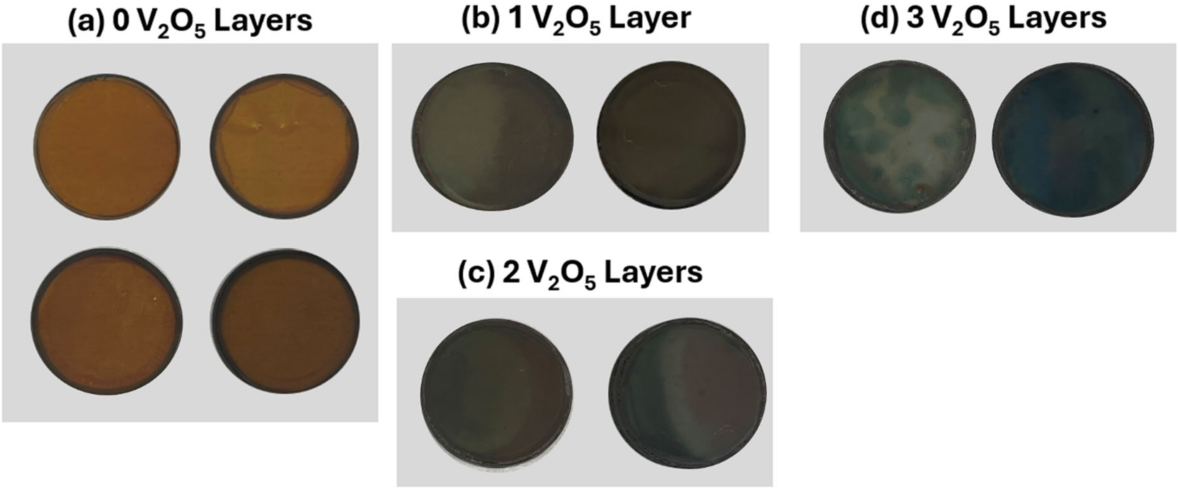

Figure S4. Optical images of the VO_2 on Si samples used for the VO_2 on V_2O_5 study sorted by the total number of V_2O_5 layers on the sample before adding the VO_2 layer on top. This range includes 0 layers (a), one layer (b), two layers (c), and three layers (d).

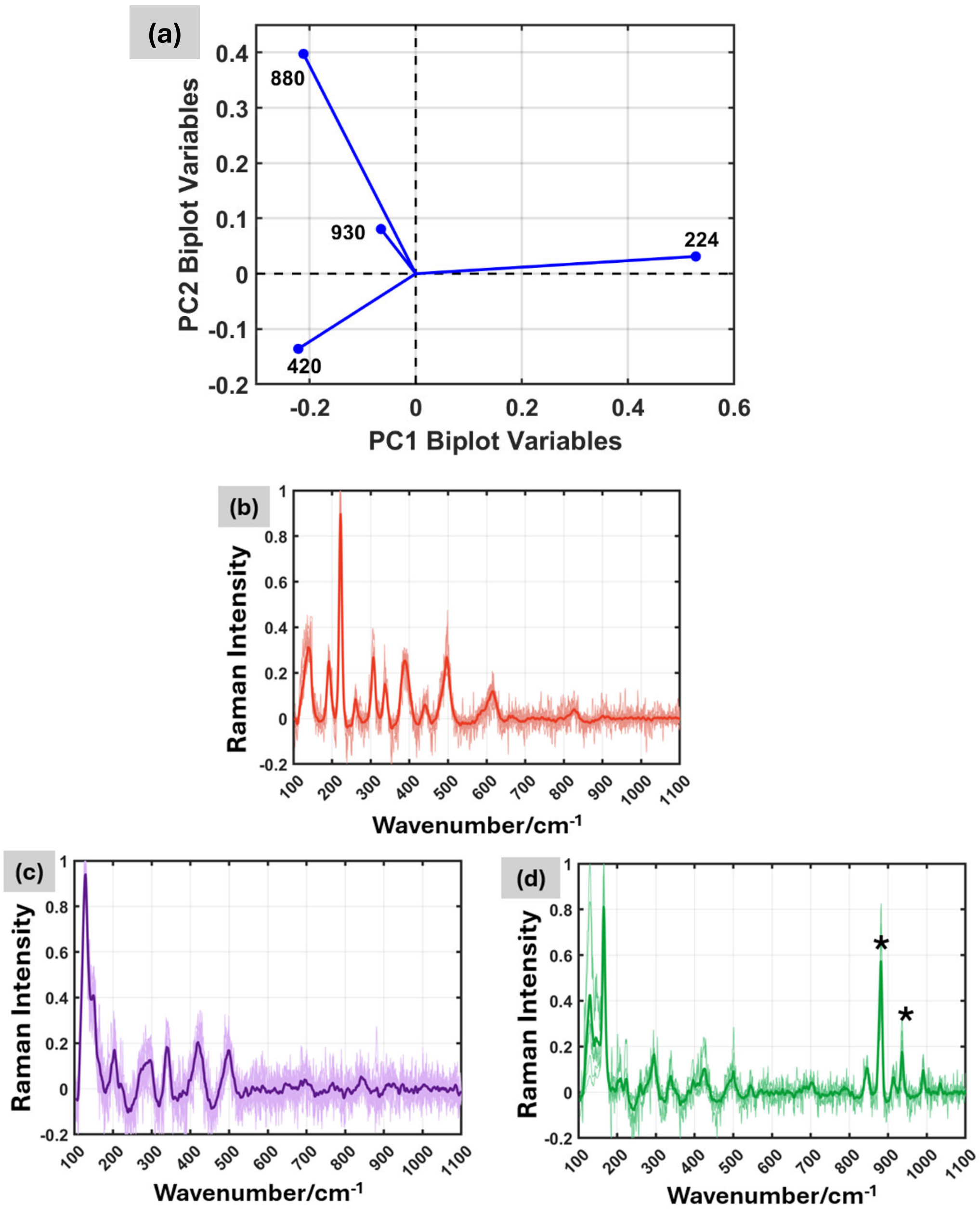

Figure S5. The biplot corresponds to the PCA plot shown in **Figure 5** (a). The biplot was filtered to show only the most influential variables (Raman peaks) in the PC1 and PC2 direction. The Raman spectra of the samples sorted by the three primary PCA groups are shown: VO_2 (b), V_6O_{13} (c), and V_6O_{13} + V_3O_7 (d). The asterisks in (d) denote influential peaks at 880 cm^{-1} and 930 cm^{-1} driving the group along the positive PC2 axis.

Sample #	Heat Treatment
Sample #1	350 °C 0.75 hours
Sample #2	350 °C 7 hours
Sample #3	228 °C 5.5 hours +<br>350 °C 3 hours
Sample #4	228 °C 5.5 hours +<br>350 °C 65.5 hours
Sample #5	300 °C 2.75 hours
Sample #6	300 °C 4.25 hours
Sample #7	300 °C 6.25 hours
Sample #8	300 °C 7.25 hours
Sample #9	300 °C 7.25 hours
Sample #10	300 °C 7.25 hours

Table S2. Thermal treatment recipes for the 10 vanadium samples.

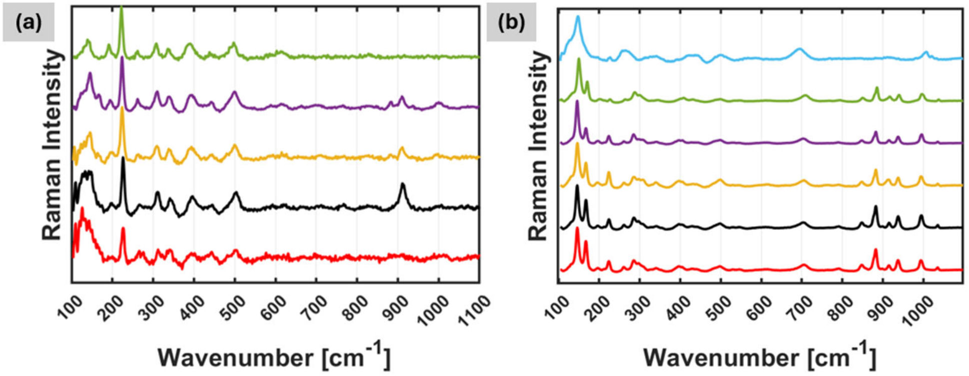

Figure S6. Raman spectra of the thermally treated Vanadium samples. The short aged samples (a) feature a VO_2 spectra (with the VO_2 reference shown first) with V_3O_7 present in some samples and the long aged samples (b) are mostly a mix of V_2O_5 and V_3O_7.

This work was performed under the auspices of the U.S. Department of Energy by Lawrence Livermore National Laboratory under Contract DE-AC52-07NA27344. LLNL release number: LLNL-JRNL-2015562.
